# Revealing the Critical Regulators of Modulated Smooth Muscle Cells in Atherosclerosis in Mice

**DOI:** 10.3389/fgene.2022.900358

**Published:** 2022-05-23

**Authors:** Wenli Zhou, Yongyi Bai, Jianqiao Chen, Huiying Li, Baohua Zhang, Hongbin Liu

**Affiliations:** ^1^ Medical School of Chinese PLA, Beijing, China; ^2^ Department of Cardiology, The Second Medical Center, Chinese PLA General Hospital, Beijing, China; ^3^ Department of Health Care, The Second Medical Center, Chinese PLA General Hospital, Beijing, China

**Keywords:** atherosclerosis, smooth muscle cells, phenotypic modulation, single-cell RNA sequencing, gene regulatory network

## Abstract

**Background:** There are still residual risks for atherosclerosis (AS)-associated cardiovascular diseases to be resolved. Considering the vital role of phenotypic switching of smooth muscle cells (SMCs) in AS, especially in calcification, targeting SMC phenotypic modulation holds great promise for clinical implications.

**Methods:** To perform an unbiased and systematic analysis of the molecular regulatory mechanism of phenotypic switching of SMCs during AS in mice, we searched and included several publicly available single-cell datasets from the GEO database, resulting in an inclusion of more than 80,000 cells. Algorithms implemented in the Seurat package were used for cell clustering and cell atlas depiction. The pySCENIC and SCENIC packages were used to identify master regulators of interested cell groups. Monocle2 was used to perform pseudotime analysis. clusterProfiler was used for Gene Ontology enrichment analysis.

**Results:** After dimensionality reduction and clustering, reliable annotation was performed. Comparative analysis between cells from normal artery and AS lesions revealed that three clusters emerged as AS progression, designated as mSMC1, mSMC2, and mSMC3. Transcriptional and functional enrichment analysis established a continuous transitional mode of SMCs’ transdifferentiation to mSMCs, which is further supported by pseudotime analysis. A total of 237 regulons were identified with varying activity scores across cell types. A potential core regulatory network was constructed for SMC and mSMC subtypes. In addition, module analysis revealed a coordinate regulatory mode of regulons for a specific cell type. Intriguingly, consistent with gain of ossification-related transcriptional and functional characteristics, a corresponding small set of regulators contributing to osteochondral reprogramming was identified in mSMC3, including Dlx5, Sox9, and Runx2.

**Conclusion:** Gene regulatory network inference indicates a hierarchical organization of regulatory modules that work together in fine-tuning cellular states. The analysis here provides a valuable resource that can provide guidance for subsequent biological experiments.

## Introduction

Atherosclerosis (AS) of the main artery (e.g., coronary artery and carotid artery, etc.) in the human body is one of the major potential health killers among the elderly (Stary et al., 1995; Roth et al., 2020). On one hand, the enlargement of the lesion size can directly lead to vascular stenosis and reduce the blood supply; on the other hand, the rupture of vulnerable plaques can lead to local or distal thrombosis, resulting in serious clinical events such as cerebral infarction or myocardial infarction which depends on the location of the atherosclerotic plaques (Basatemur et al., 2019). Although current lifestyle and clinical drug interventions such as lowering blood lipids and controlling blood pressure have worked well in the prevention and prognosis improvement of cardiovascular diseases associated with AS (Baigent et al., 2010; Shah et al., 2020), we have to admit that there are still large residual risks that are needed to be resolved (Geovanini and Libby, 2018).

Vascular smooth muscle cells (SMCs) and SMC-derived cells contributed a lot to the cell heterogeneity in AS lesions. Through integrating stimulation by a variety of microenvironmental factors, SMCs migrate from the media to the inner membrane and undergo transcriptional reprogramming (usually designated as phenotypic modulation), which is characterized by losing their classic contractile function and increasing the ability to proliferate and synthesis (Davis-Dusenbery et al., 2011; Allahverdian et al., 2014). The role of phenotypically modulated SMCs in AS may be beneficial or detrimental, depending on their location in the lesion. For example, SMCs located in the fibrous cap can increase its thickness through synthesizing fibers, thereby promoting plaque stability (Basatemur et al., 2019), while SMCs located inside the plaque are one of the major sources of foamy cells and contribute to the formation of a necrotic core, thereby promoting the progression of AS lesions (Allahverdian et al., 2014; Feil et al., 2014). Interventive strategies aiming at regulating the phenotype of SMCs may help to change the properties of plaques, which is, indeed, implicated by several animal experiments using knockout mice lacking specific transcription factors (TFs) (Shankman et al., 2015; Cherepanova et al., 2016; Wirka et al., 2019; Alencar et al., 2020). Intriguingly, most of the current treatment strategies for cardiovascular diseases have no direct effect on SMC phenotypic modulation. Therefore, strategies that directly target phenotypic modulation of SMCs in AS are expected to provide new therapeutic hope.

Single-cell RNA (scRNA) sequencing allows researchers to explore cell heterogeneity at the single-cell level (Saliba et al., 2014; Winkels et al., 2018; Schupp et al., 2021). The emerging publications involving scRNA sequencing have largely improved our understanding of the cellular heterogeneity both in normal arterial tissue and atherosclerotic lesions, especially the studies performed by combined use of scRNA sequencing and SMC-lineage tracing technology (Fernandez et al., 2019; Lin et al., 2019; Wirka et al., 2019; Ma et al., 2022). For example, both Wirka et al. (2019) and Pan et al. (2020) revealed that classical SMCs can dedifferentiate into fibroblast-like phenotypes but showed a slightly different view about the potential of SMC’s transdifferentiation into macrophage-like cells. After all, there are still some questions to be resolved: What are the underlying regulatory mechanisms that maintain different phenotypic subtypes of SMCs? What TFs constitute the core regulatory network? How do different TFs coordinate together to determine a specific phenotype of SMCs? Notably, solving these phenotypic modulation-related regulatory questions will help us develop therapeutic strategies targeting SMCs with important clinical implications. Fortunately, the explosive accumulation of scRNA sequencing data has given birth to many efficient and reliable algorithms for gene regulatory network (GRN) inference, such as SCENIC (Aibar et al., 2017; Van de Sande et al., 2020). In our current study, we first performed an integrative bioinformatic analysis to construct the cellular landscape of atherosclerotic lesions; based on this well-characterized cell atlas, we further systematically depict a comprehensive GRN atlas of phenotypically modulated SMCs in atherosclerotic lesions in mice.

## Methods

### Preparation of Publicly Available Datasets

To ensure our analysis performance, we included publicly available single-cell datasets which meet the following criteria (Roth et al., 2020): the raw UMI matrix can be obtained from GEO or Single Cell Portal database (Stary et al., 1995); the genotype of mice used for dataset production was wild type, Apoe−/− or Ldlr−/− on a background of G57BL/6J (Basatemur et al., 2019); the tissue used was dissected from the aorta, and the cDNA libraries were constructed on the 10X genomics platform. After searching and filtering, the following datasets are included: GSE117963 (Dobnikar et al., 2018), GSE131776 (Wirka et al., 2019), GSE131777 (Wirka et al., 2019), GSE155513 (Pan et al., 2020), and GSE174384 (Kalluri et al., 2019). The UMI matrix was downloaded for further analysis. Details about the included datasets are shown in Supplementary Table S1. Notably, cell lineage information was provided in most of the included datasets.

### Integrative Analysis of Single-Cell RNA-Seq Data

Only cells of high quality were kept for integrative bioinformatics analysis. To be specific, cells with genes no less than 200 and percent of mitochondrial genes no more than 20% were retained. Then, the UMI matrix was preprocessed using Seurat’s SCTransform function (Hafemeister and Satija, 2019) for further downstream analysis. Here, top 3,000 highly variable features were returned and stored in the SCT assay. Then, the transformed data were further integrated using the RunHarmony function (Korsunsky et al., 2019) with default parameters to correct the batch effect, followed by dimension reduction and clustering analysis. To be specific, UMAP (McInnes et al., 2018) was selected to visualize single cells in the reduced two-dimensional space, and cells were clustered into different populations using a graph-based and modularity optimization-based clustering algorithm (Waltman and van Eck, 2013) implemented in the Seurat package. Once clustered, each cell group was assigned a biological name with comprehensive utilization of known information, including classic cell-type-specific markers, cell-lineage information, and disease status of tissue (atherosclerosis or normal tissue).

### Differential Expression and Functional Enrichment Analysis

Differentially expressed genes (DEGs) for each cell group were identified using FindAllMarkers in the Seurat package with the Wilcoxon test. Here, the threshold of log-fold change was set at 0.25. Gene Ontology (GO) enrichment analysis was performed using clusterProfiler, and redundant GO terms were removed using simplify function with a cutoff of 0.7.

### Regulon Inference and Activity Evaluation

To comprehensively depict GRNs, the expression profile of more than 80,000 cells in total was selected as input for SCENIC. Also, the analysis was performed using pySCENIC (corresponding to RcisTaret 1.2.0 and AUCell 1.4.1) followed the pipeline described in Van de Sande et al. (2020).

### Regulon Specific Score

To identify master regulons, we calculated cell-type specificity scores for each regulon in every cell type using the RSS method implemented in *calcRSS* function in the R package SCENIC. RSS algorithms were developed based on Jensen–Shannon divergence by Suo et al. (2018) and have been proved to be an effective method to identify cell-type-specific regulons. Specifically, regulons with the highest cell-type-specific scores were considered candidates for essential regulators.

### Regulon Module Analysis

To have an overview of similarity and explore the potential coordination mode between regulons, regulon module analysis was performed using the method described by Suo et al. (2018). In detail, Pearson correlation coefficients (PCCs) between regulons were first evaluated based on the activity score matrix and then taken as input for calculating connectivity specify index (CSI) according to the equation:

$$CSI_{A,B}=\frac{n\;\;\left(number\;of\;regulon\;pairs\;with\;a\;PCC<PCC_{A,B}\right)}{N\;\left(total\;number\;of\;regulon\;pairs\right)}.$$

Second, regulon modules were identified using unsupervised hierarchical clustering based on Euclidean distance calculated using the CSI matrix.

### Data Visualization

All visualizations were performed in R. Specifically, UMAP was visualized by using Seurat. The violin plot, bar plot, and line graph were visualized using ggplot2. Heatmap was visualized using ComplexHeatmap or pheatmap. Networks were constructed using Cytoscape (v3.8.2).

## Results

### An Overview of Cell Heterogeneity in Atherosclerotic Plaques in Mice

**FIGURE 1:**
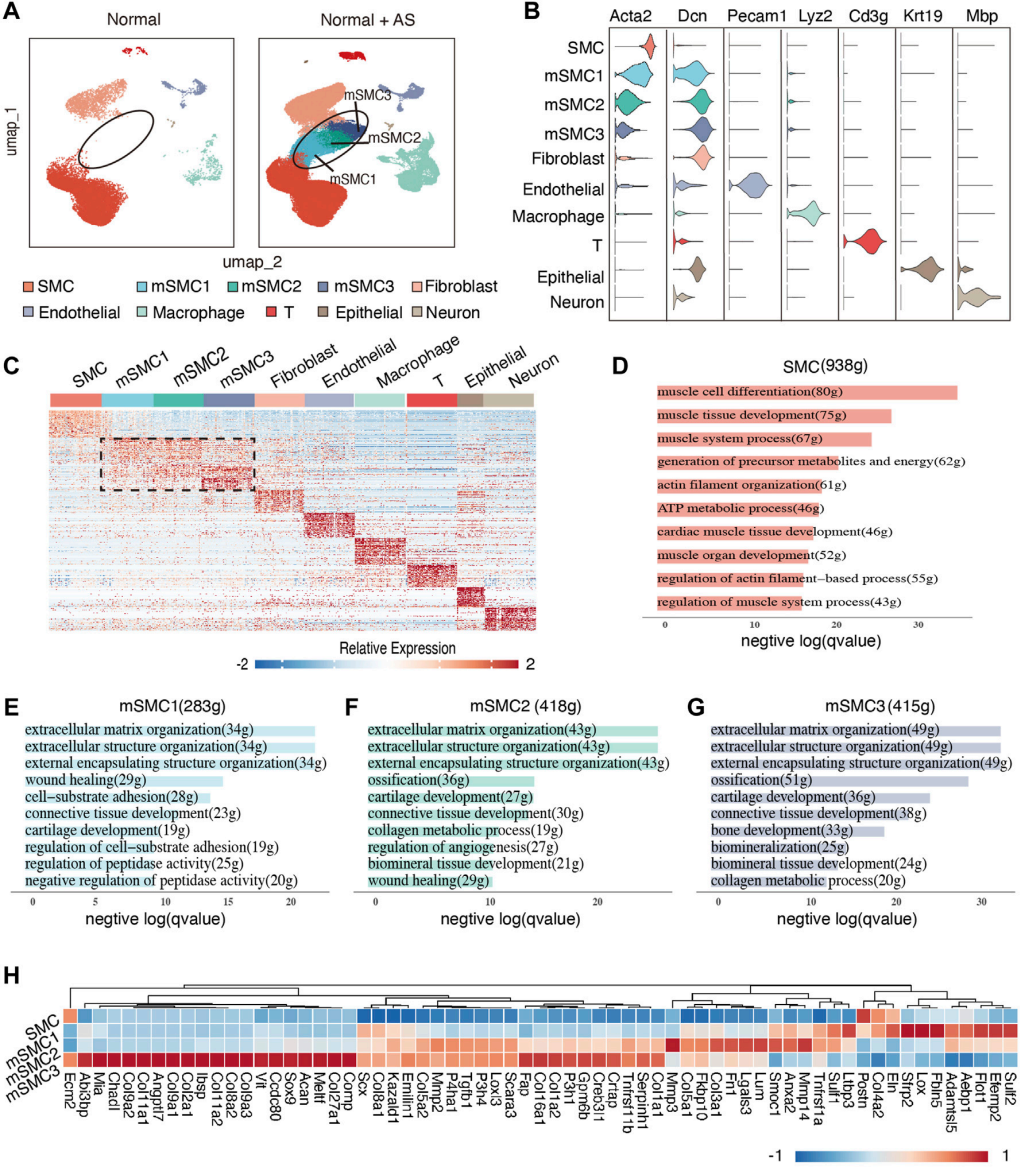
Identification of cell types and functional characterization of each cell type in plaques in mice. **(A)** UMAP visualization of all cells (right) and cells from normal tissue (left). Normal tissue is defined as aorta dissected from wild-type mice treated with a chow diet. **(B)** Violin plot of representative classic markers for each identified cell type. **(C)** Heatmap of top 100 DEGs for each cell type. Scaled log-normalized data were used for visualization. **(D–G)** Top 10 enriched GO biological process terms for SMC, mSMC1, mSMC2, and mSMC3, respectively. The number of DEGs for each cell type and involved genes for each GO term was designated in the brackets with g-representing genes. **(H)** Heatmap of genes enriched in an extracellular matrix organization in mSMC subtypes. Scaled log-normalized data were used for visualization. SMCs, smooth muscle cells; mSMCs, phenotypically modulated SMCs; DEGs, differentially expressed genes.

A large dataset facilitates data mining for more valuable hidden information. Hence, we first yielded a huge dataset consisting of 84,048 cells of high quality by including several appropriate scRNA datasets in public databases (see *Methods*). Among all the cells, 17% cells are from normal aortic tissue dissected from aorta tissue of wild-type C57BL/6J mice, and 55.1% cells are clearly defined as SMC-derived cells through lineage-tracing methods (Supplementary Figure S1), enabling us to identify disease-related cell populations and define the cell lineage origin. Then, a reliable annotation process was performed. First, through taking advantage of well-established cell type-specific markers, seven main cell types in total were identified in normal samples, including SMCs, fibroblasts, macrophages, endothelial cells, T cells, epithelial cells, and neurons (Figures 1A,B; Supplementary Figure S2). Second, a comparison of clustering results in the UMAP-generated two-dimensional space displayed that those cells in three clusters (Davis-Dusenbery et al., 2011; Shankman et al., 2015; Basatemur et al., 2019) were only accumulated in atherosclerotic lesions, and furthermore, known cell lineage information exhibited that these newly emerging cell phenotypes with AS development were SMC-derived (Supplementary Figure S3). Here, these three clusters were designated as phenotypically modulated SMCs with mSMC1, mSMC2, and mSMC3 corresponding to cluster3, cluster8, and cluster10, respectively (Figure 1A). Intriguingly, these mSMCs displayed a gradually reduced expression of known SMC markers, while a gradient increased expression of fibroblast markers along mSMC1 to mSMC3, which is consistent with the reported observation that SMCs transitioned to a fibroblast-like phenotype in atherosclerotic lesions.

### Functional Characterization of SMC-Derived Cell Subtypes in Atherosclerotic Lesions

High cellular heterogeneity drives the formation of complex disease-associated microenvironments. To have an overview understanding of functional roles of each cell type in AS lesions, we first identified the top DEGs that distinguish them from each other using the Seurat package (Figure 1C; Supplementary Table S2). DEG analysis revealed that 938 genes were relatively enriched in SMCs and as expected, these highly expressed genes were mainly enriched in muscle cell differentiation, muscle tissue development, and actin filament organization, etc., as revealed by GO enrichment analysis (Figure 1D; Supplementary Table S3). By contrast, mSMC subtypes exhibited relatively specific transcriptional profiles and unique corresponding functional characteristics (Figures 1E–G; Supplementary Tables S4–S6). To be specific, upregulated genes in mSMC1 were mainly involved in several biological pathways regulating cellular adhesion behavior (e.g., cell-substrate adhesion and regulation of cell-substrate adhesion) (Figure 1E), which is important for non-resident cells’ accumulation in the intima (e.g., SMCs and macrophages); also, gene signatures of mSMC3 primarily enriched in the ossification-related process (e.g., regulation of ossification, bone development, and cartilage development, etc.) (Figure 1G), supporting the reported osteochondral transdifferentiation. As for mSMC2, both transcriptional and functional results supported a transitional status between mSMC1 and mSMC3 (Figures 1C,F) which is further supported by the developmental trajectory constructed by Monocle2 (Supplementary Figure S4). In detail, cells in the mSMC3 cluster were concentrated at the end of the trajectory, representing a terminally phenotypically modulated mSMC subtype, while mSMC1 and mSMC2 distributed along the trajectory between SMC and mSMC3, indicating that phenotypic switching of SMCs occurring in a continuous model. Functional enrichment analysis was also performed for other main cell types with results suggesting an altered immune process in AS lesions (Supplementary Figure S5). Taken together, cell types in AS lesions are of high heterogeneity, and each cell type plays a unique role with terminally phenotypically modulated SMC facilitating calcification of AS lesions.

In addition, we noticed that all mSMC subtypes played an active role in ECM remodeling (Figures 1E–G) which is consistent with the well-known opinion that SMC-derived cells contributed to ECM formation at all stages during AS progression (Ponticos and Smith, 2014; Langley et al., 2017). Considering that ECM components play significant roles in determining plaque’s fate, we thus further explore whether there exist differences between genes that were responsible for ECM remodeling in mSMC subtypes. Results showed that mSMC1 displayed the highest expression level of Sfrp2 (Figure 1H; Supplementary Figure S6), the protein encoded which has been reported to be similar to the cysteine-rich domain of Frizzled G-protein-coupled receptor and thus considered to function as Wnt inhibitors through binding Wnts (Logan and Nusse, 2004). Notably, the Wnt singling pathway is pleiotropic, with effects on regulating the function of ECs, macrophages, and SMCs in AS (Gay and Towler, 2017; Matthijs Blankesteijn and Hermans, 2015). However, whether activation of Wnt signals manifests beneficial or detrimental effects depends on the complex microenvironment, and Sfrps may exhibit a promotive role in Wnt signals through some mechanisms, such as decreasing Wnts degradation or facilitating Wnt secretion (Logan and Nusse, 2004). Hence, further research is needed to elucidate the specific role of Sfrp2’s upregulation. In addition, mSMC1 showed relatively high expression of several other genes involving the VEGF signaling pathway (e.g., Sulf2), TGF-β signaling pathway (e.g., Ltbp3), and TNF singling pathway (e.g., Tnfrsf1α) (Figure 1H; Supplementary Figure S6). Together with the functional enrichment results, it is reasonable for us to conclude that the mSMC1 phenotype is induced under pro-atherosclerosis stimuli and in turn contributes to a signaling system that may favor cell survival and proliferation. As for mSMC3, cells mainly displayed active synthetic activity in ossification-related proteins, including various types of collagens and non-collagen proteins (Figure 1H; Supplementary Figure S6). For example, the protein encoded by Ibsp, typically secreted by bone-related cell types, is a major non-collagen protein of bone and has been reported to be upregulated in human AS plaques (Ayari and Bricca, 2012); protein encoded by Acan is reported to constitute cartilage tissue and primally plays a role in resisting pressure (Chang et al., 2019). These data indicate that the osteochondral program is activated among mSMC3 cells. As is known, ECM degradation is one of the key steps of ECM remodeling which is primarily mediated by matrix metalloproteases (Mmps) (30). Thus, the observed increased expression of several Mmps (including Mmp2, Mmp3, and Mmp14) supported the significant role of mSMCs in ECM remodeling, thus regulating AS progression.

Taken together, with the development of atherosclerosis, disease-specific SMC-derived cells accumulated in lesions and exhibited sequential temporal changes in transcriptional and functional profiles, which is closely associated with the severity of atherosclerosis, together indicating a crucial role of mSMCs in plaque development.

### Construction of Gene Regulatory Networks in SMCs and mSMCs in Atherosclerosis

The transcriptional profile is largely determined by the regulation of several key TFs. So, which TFs participate in the formation and maintenance of the transcriptional characterizations in different mSMC phenotypes? To solve this problem, we performed GRN inference by taking advantage of computational algorithms implemented in pySCENIC (Van de Sande et al., 2020), a tool that improves reference reliability by utilization of cis-regulatory motif enrichment analysis in the pipeline. Results showed that 277 identified regulons and 237 regulons with high credibility were kept for further exploration, resulting in an involvement of 8,522 genes in total and a range of 3–2,188 genes for an individual regulon (Supplementary Table S7). These identified regulons provided us an opportunity to investigate the core regulatory network for each well-characterized cell identity.

**FIGURE 2:**
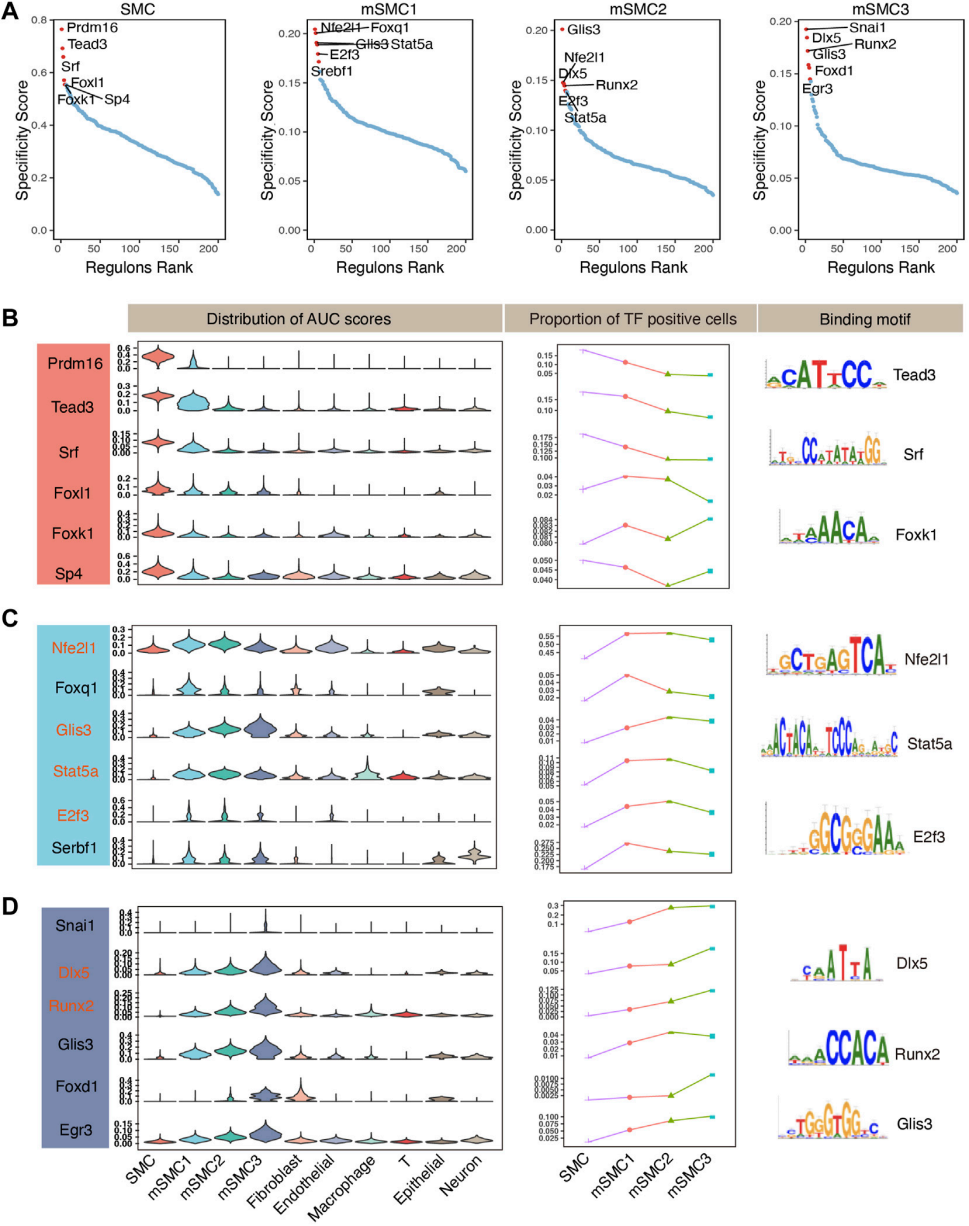
Construction of gene regulatory networks in SMC-derived cells in atherosclerosis in mice. **(A)** Identified regulons were ranked based on their cell-type specificity scores in SMCs and mSMC subtypes. Key TFs of the top six specific regulons for each cell type were designated. **(B)** Left panel shows distribution of AUC scores of the top six specific regulons for SMCs. The panel in the middle represents the proportion of corresponding TF-positive cells in SMCs and mSMC subtypes. The right panel displays binding motifs for representative TFs for SMCs. **(C)** Same as **(B)** but for mSMC1. **(D)** Same as **(B)** but for mSMC3. Considering mSMC2 shares top specific regulons with mSMC1 or mSMC3, the top 6 mSMC2-specific regulons were highlighted in light red in **(C,D)**. SMCs, smooth muscle cells; mSMCs, phenotypically modulated SMCs; DEGs, differentially expressed genes; AUC, area under the curve.

Next, based on activity scores, we defined a regulon specificity score (RSS) for each regulon in each cell type (Supplementary Table S8), and regulons with the highest RSS values were defined as master regulators for corresponding cell identity. Notably, RSS has been proved to be an effective approach in distinguishing crucial regulators for a well-characterized cell type (Suo et al., 2018). Here, we focus on recognizing master regulons for SMCs and mSMC subtypes. Results of our network analysis displayed that the most specific regulons in SMCs including Prdm16(+), Tead3(+), Srf (+), Foxl1(+), Foxk1(+), and Sp4(+), which is supported by the relatively higher activity scores across cells in SMCs (Figures 2A,B). Of note, Srf (serum response factor), a member of MADS-box family of TFs (Shore and Sharrocks, 1995), has been well established as an indispensable regulator in maintaining SMC identity through binding to conserved CArG boxes located in promoter regions of nearly all the SMC marker genes (McDonald et al., 2006). In addition, Foxk1, a member of the Forked family, can interact with Srf and thus modulate the expression pattern of Srf-dependent genes (Abid et al., 2005; Freddie et al., 2007). Tead3 is a YAP downstream effector, and Han ZB’s team proved that the Yap/Tead3 signaling pathway played a significant role in cardiovascular lineage commitment (Almontashiri et al., 212015; Han et al., 2020). Taken together, these observations obtained from the literature mining strongly validated the reliability of our GRN inference analysis.

As for mSMC1, Nfe2l1 regulon scored the highest, followed by Foxq1, Glis3, Stat5a, E2f3, and Srebf1, indicating these TFs may constitute the core gene regulatory network for the mSMC1 phenotype (Figures 2A, C). Indeed, Foxq1 has been reported to downregulate the expression of muscle cell-specific genes by repressing the activity of corresponding promoters, and it played an antiapoptotic role in cancer tissue (Kaneda et al., 2010). Intriguingly, several calcification-related TFs, including Runx2 (Komori, 2002; Byon et al., 2008) and Dlx5 (Shirakabe et al., 2001; Lee et al., 2005) (Figures 2A,D), showed the highest RSS values in mSMC3, which is consistent with the functional repertoire as revealed by GO analysis (Figure 1G). As additional supportive evidence, activity scores of the top-ranked master regulons for each cell identity were relatively higher than in other cell phenotypes, and these candidate master TFs were expressed in a higher proportion of cells within the corresponding cell population, especially in SMCs and terminally modulated mSMC3 (Figures 2B–D). Interestingly, cells in mSMC2 shared the most specific regulons with mSMC1 and mSMC3 (Figure 2A). This phenomenon, to a certain degree, can be attributed to its transitional role in phenotypic modulation from SMCs to mSMC3 as revealed by trajectory analysis (Supplementary Figure S4). Taken together, our gene regulation network analysis prioritized several reliable regulons as candidate crucial regulators for SMCs and SMC-derived cell phenotypes.

### Module Analysis of Identified Regulons in Atherosclerosis in Mice

**FIGURE 3:**
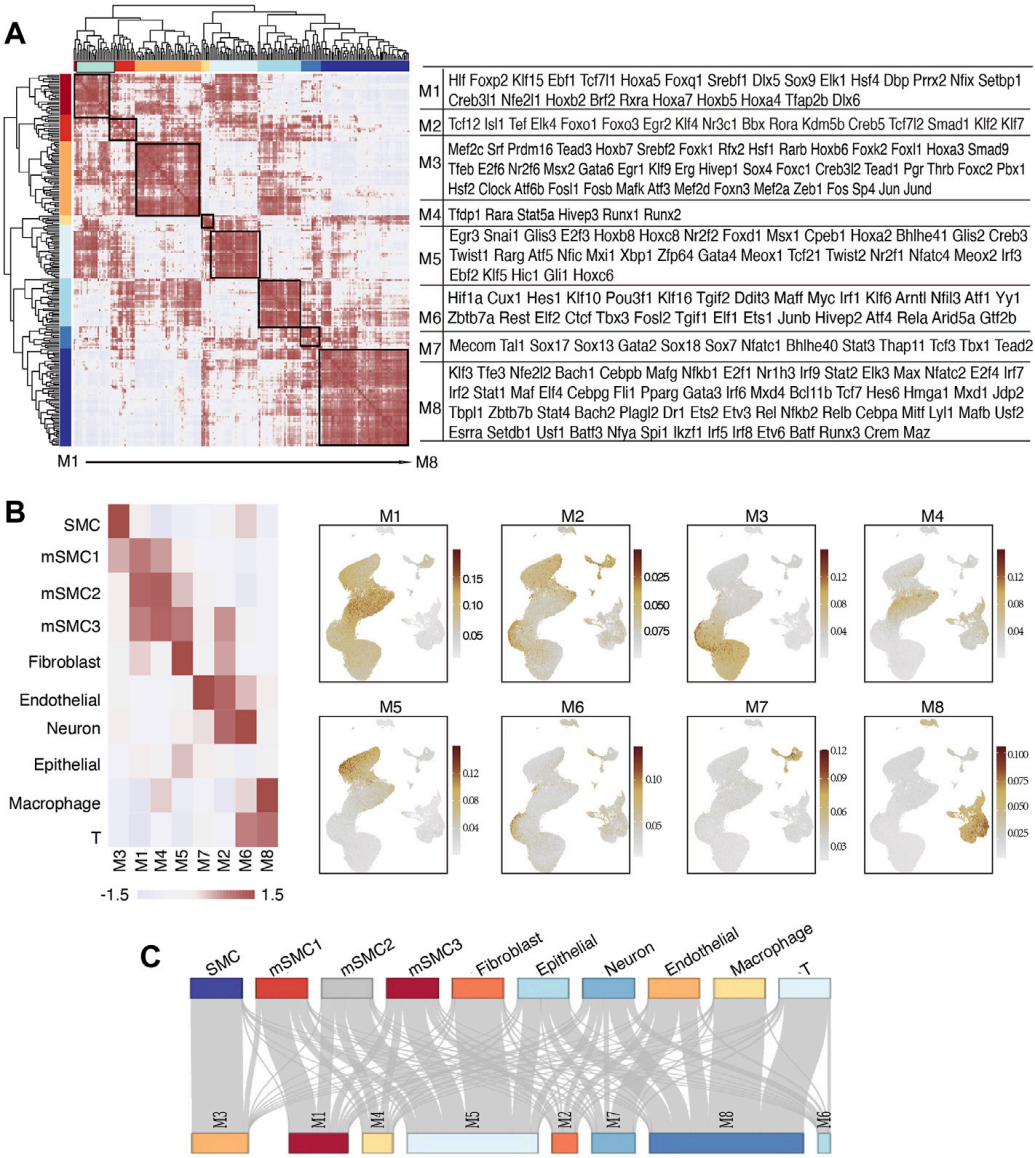
Module analysis of identified regulons in atherosclerosis in mice. **(A)** Heatmap displays clustered regulon modules based on the CSI matrix along with the included regulons being shown in the right. **(B)** Heatmap in the right panel shows the average activity scores of each identified module in each cell type. The left panel displays the UMAP visualization of the average scores of eight identified modules in each single cell. **(C)** Sankey plot demonstrates the distribution pattern of top 20 specific regulons for each cell type in the representative regulon modules. CSI, connection specificity index.

Considering the biological consensus that TFs often cooperatively regulate transcriptional profiles in a coordinate mode, we performed module analysis by sequentially calculating activity scores, Pearson correlation coefficient, and connection specificity index (CSI). Through unsupervised hierarchical clustering, all the regulons were organized into eight modules, namely module 1 to module 8 (M1-M8) (Figure 3A; Supplementary Figure S7). We noticed that top6 SMC-specific regulons were all clustered into M3, which supported the hypothesis that regulons in M3 may coordinately regulate the differentiation fate of SMCs. To further support this, we observed that M3 also included several regulons, the associated TFs of which have been reported to play a role in determining muscle cell differentiation, such as Mef2 isoforms (Shore and Sharrocks, 1995; Spicer et al., 1996). Similar clustering patterns were also found in other cell types, such as fibroblast (M5), endothelial cells (M7), macrophages (M8), and T cells (M8). For example, among regulons in M8, Cebpb (Ruffell et al., 2009) has been well described as an essential regulator for macrophages, and Nfatc2 (Peng et al., 2001) is reported to play a key role in T cells. As for mSMC subtypes, top-ranked regulons based on RSS scores were primarily distributed in M1, M4, and M5. According to these findings, we hypothesized that the identity of a specific cell group was mainly determined by a corresponding regulon cluster. We calculated the average activity score of each module in each cell type (Figure 3B), and the heatmap showed that indeed, a specific module was mainly activated in one or two cell types and vice versa. Intriguingly, consistent with the continuous transition mode as revealed by transcriptional and functional repertoires of mSMCs, a similar mode was observed in the regulatory network (Figure 3B). In addition, mSMC-associated modules (namely, M1, M4, and M5) included several reported ossification- or calcification-related TFs, including Dlx5 (Shirakabe et al., 2001), Sox9 (Augstein et al., 2018), Runx2 (Byon et al., 2011), Rarg (Pan et al., 2020), and Twist1 (Rice et al., 2000; Bialek et al., 2004) Finally, we explored the distribution model of the top 20 cell-type-specific regulons for each cell type in eight modules and represented in a Sankey plot (Figure 3C). The results were showed for a specific cell type, and the top 20 regulons concentrated primarily in a module (e.g., top 20 regulons of SMCs in M3). Taken together, it is reasonable for us to conclude that a cell type’s fate is determined by a core regulatory network and simultaneously is regulated by some coordinate TFs, which is of vital significance for researchers to develop re-programming strategies for mSMCs.

## Discussion

Phenotypic modulation of SMCs is a key biological process during atherosclerotic plaque formation. Considering the high proportion of SMC-derived cells and their crucial roles in inflammation and calcification, targeting phenotypic modulation of SMCs can be a promising strategy to slow down the progression of AS or increase the stability of the existed plaques. Indeed, such strategies have yielded satisfactory experimental results by knocking down a TF, such as KLF4 (Shankman et al., 2015). However, there is still a lack of systematic and unbiased knowledge on the molecular regulatory mechanism for SMC phenotypic modulation. In this study, we made an in-depth exploration and detailed description of the regulatory landscape of SMC phenotypic modulation by taking the full advantage of publicly available scRNA-sequencing datasets of high quality and performing reliable bioinformatic analysis.

First of all, we depicted an overview landscape of cell types involved in AS, especially SMC-derived cell populations. With atherosclerotic lesion progression, resident SMCs undergo phenotypic modulation in a continuous style instead of binary mode as indicated by dimension reduction analysis and pseudotime analysis. As has been previously reported (Pan et al., 2020), cells belonging to the end-stage of SMC phenotypic modulation possess a phenotype that shared the most expressional and functional characteristics with fibroblasts among all the identified cell types in the lesions. Earlier phenotypically modulated SMCs were characterized by the gain of synthetic function, which contributed to a pro-inflammatory microenvironment. Though genes with much higher expression in terminally phenotypically modulated SMCs also displayed an enrichment in ECM remodeling, they are closely related to ECM ossification. Taken together, cells with varying degrees of phenotypical alterations coordinated together to form a complex disease-related microenvironment.

Next, we reliably identified the most cell type-specific regulons and their corresponding TFs, the crucial determinants of the activity of these genes in each regulon. As a well-documented regulator, Srf was among the prioritized regulators that are considered to play essential roles in maintenance of the classic SMC phenotype. Furthermore, the transcriptional profile of phenotypically modulated SMCs in the late stage tended to be determined by several calcification-related regulatory proteins, including Runx2 (Byon et al., 2011), Dlx5 (Shirakabe et al., 2001), and Sox9 (Xu et al., 2012). It is worth mentioning that analysis of the expressional and functional profiles of different SMC-lineage cells showed that terminally modulated SMCs possessed active biological behaviors involving ECM remodeling and calcification, which is well consistent with the characteristics of the constructed regulatory network. Notably, considering the fact that ossification of atherosclerotic plaques is positively associated with plaque instability and thus detrimental to the clinical prognosis (Steitz et al., 2001), the constructed regulatory network associated with ossification is of clinical significance. Finally, similar to the findings in late-stage mSMCs, early mSMCs are mainly regulated by TFs that are associated with cell proliferation and synthesis potential. Taken together, the GRNs constructed in our study possess high reliability and are of significance in research and even clinical practice in the future.

Lastly, we have to admit that there are limitations to our study. We did not dissect the regulatory network of human atherosclerotic plaques and thus did not make comprehensive comparisons in SMCs’ regulatory networks between humans and mice, which can be performed in the further data mining process.

Taken together, through our work in this study, researchers can strengthen their knowledge of SMC fate determination during AS formation in mice. More importantly, the constructed regulatory network here is of great significance. First of all, it provided an unbiased mechanistic understanding of each cell type’s transcriptional and functional profiles. Furthermore, such knowledge makes it more convenient for researchers to develop novel cell reprogramming strategies, which can be of great clinical implications. Therefore, our study provided a valuable resource for future experimental investigation.

## Data Availability

Publicly available datasets were analyzed in this study. These data can be found at: https://www.ncbi.nlm.nih.gov/geo/.
