# Supplementary material for: Revealing the Critical Regulators of Modulated Smooth Muscle Cells in Atherosclerosis in Mice
Source: Front Genet. 2022 May 23;13:900358. doi: 10.3389/fgene.2022.900358 (PMC9168464; doi:10.3389/fgene.2022.900358)
Supplement: Supplementary file 1 [file DataSheet1.PDF]

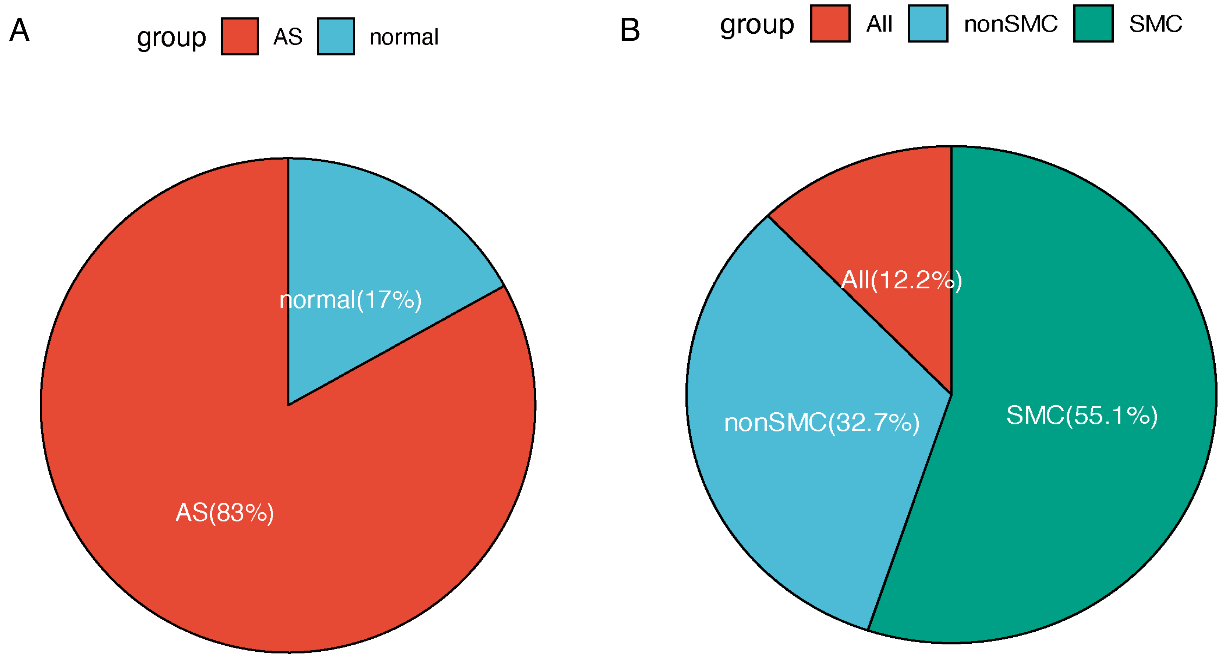

Supplementary Figure 1. Proportions of specific cell groups. A demonstrates that 17% of all included cells were separated from normal aorta tissue and 83% from atherosclerotic lesions of varying degrees. B demonstrates that 55.1% cells were clearly defined as SMC-lineage by reporter genes. SMC, smooth muscle cells.

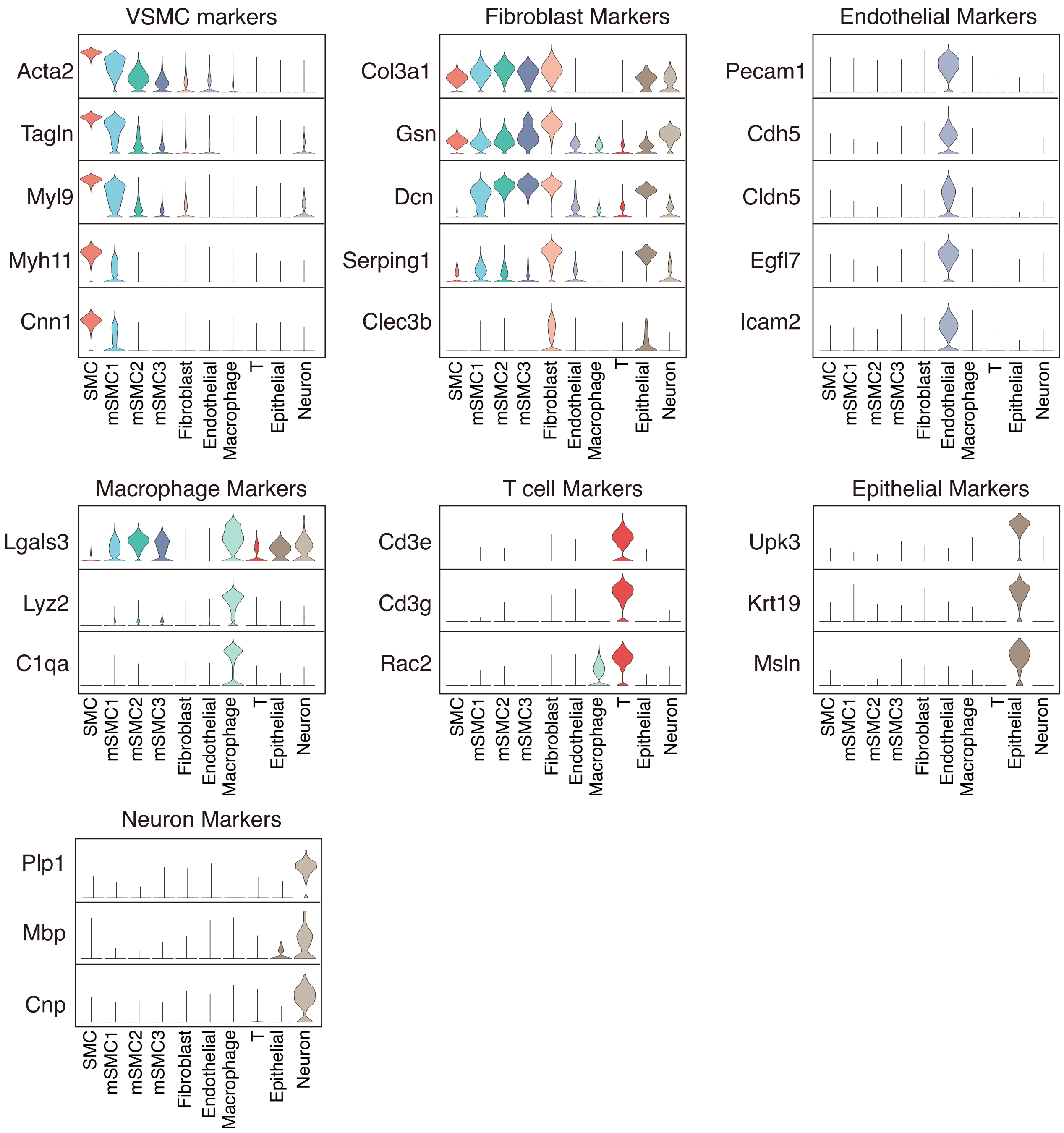

Supplementary Figure 2. Violin plot of classic marker genes for well-defined cell types in atherosclerotic lesion in mice which were used for cell annotation.

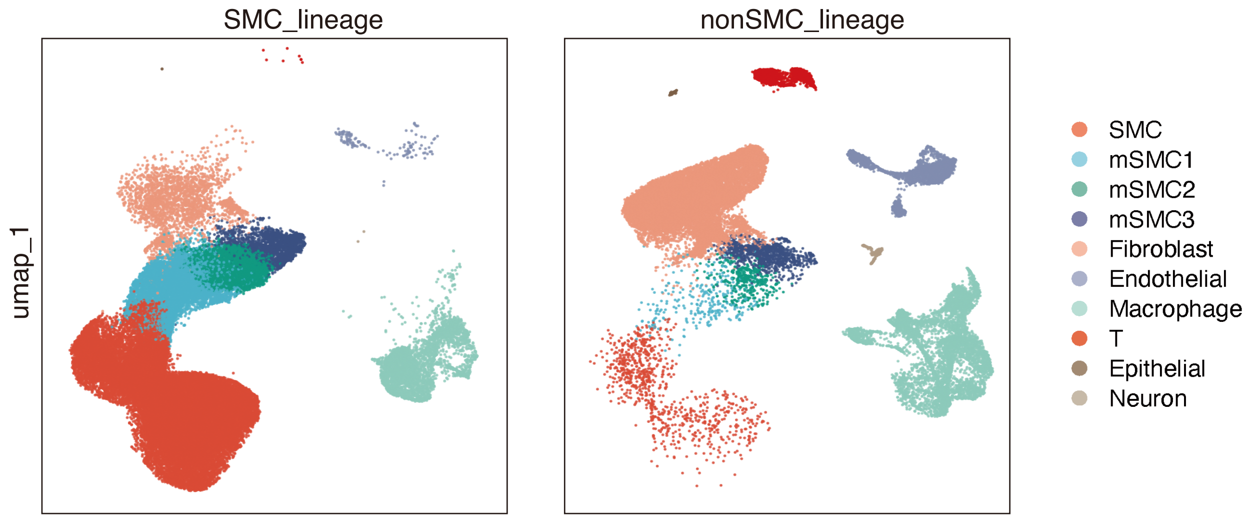

Supplementary Figure 3. Umap visualization displays the distribution of SMC-lineage and non-SMC-lineage cells in the reduced low-dimensional space. SMC, smooth muscle cells.

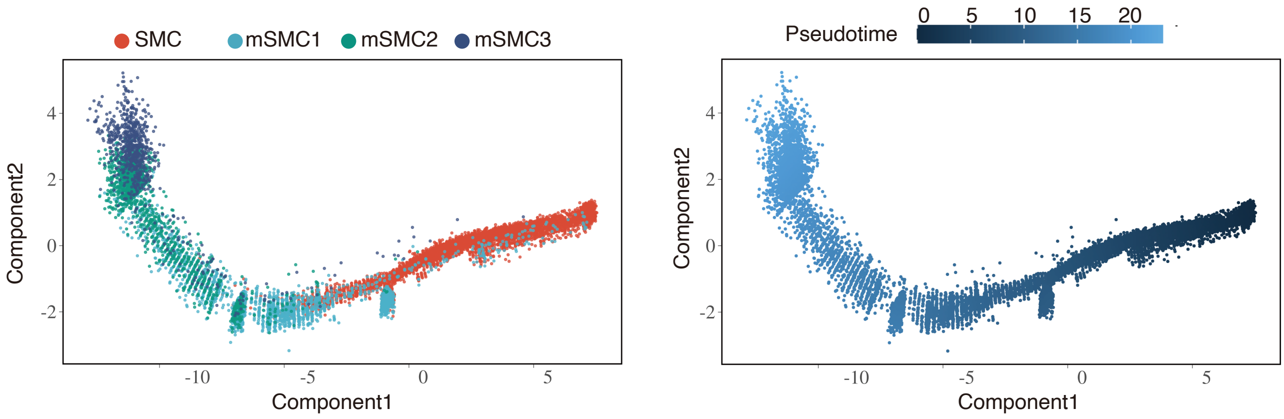

Supplementary Figure 4. Trajectory constructed using monocle2 demonstrates a continuous transition mode of SMC to mSMCs. Each dot represents a cell with colors indicating cell types in the left and indicating pseudotime in the right. SMC, smooth muscle cells. mSMC, phenotypically modulated SMCs.

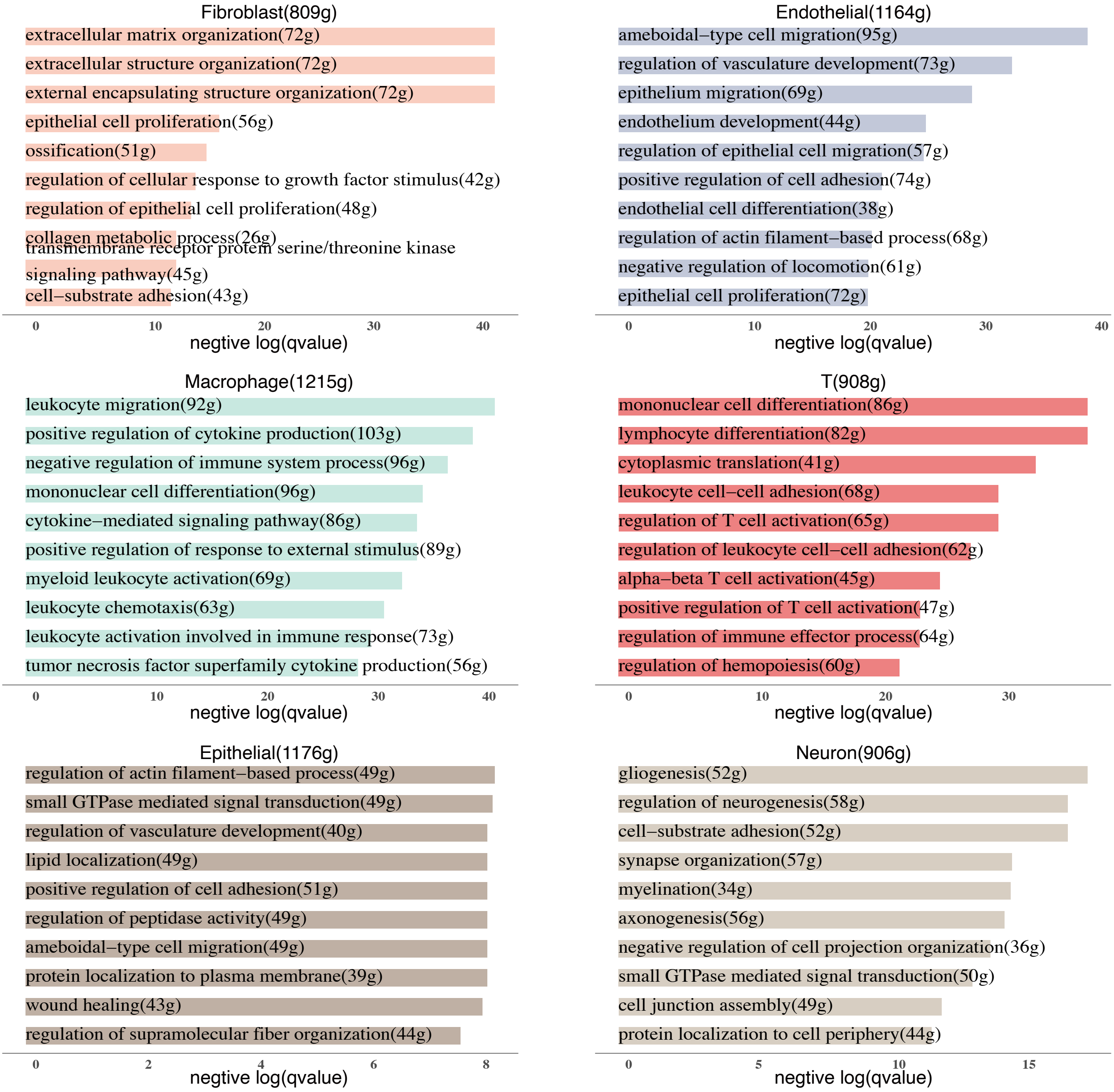

Supplementary Figure 5. Top 10 enriched GO biological process terms for fibroblasts, endothelial cells, macrophages, T cells, epithelial cells and neurons, respectively. Numbers involved genes for each GO term were designated in the brackets with g representing genes. DEGs, differentially expressed genes.

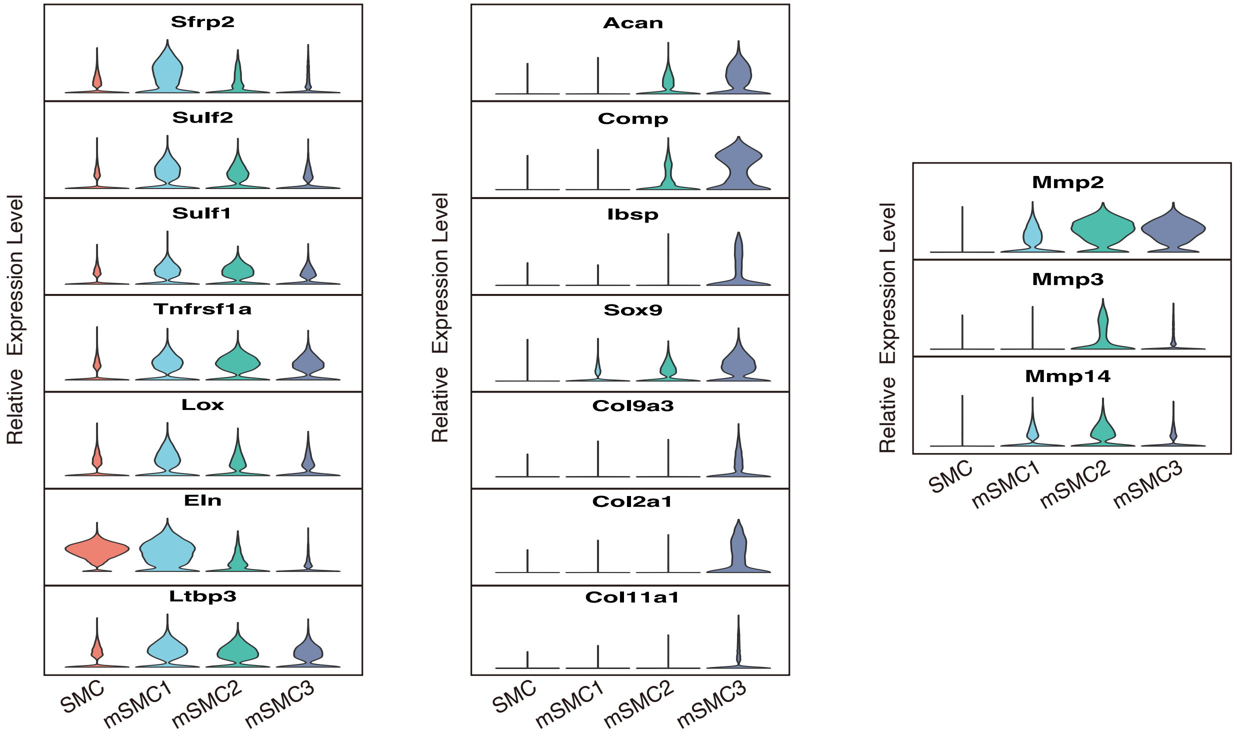

Supplementary Figure 6. Violin plot of representative genes enriched in extracellular matrix organization in mSMC subtypes.

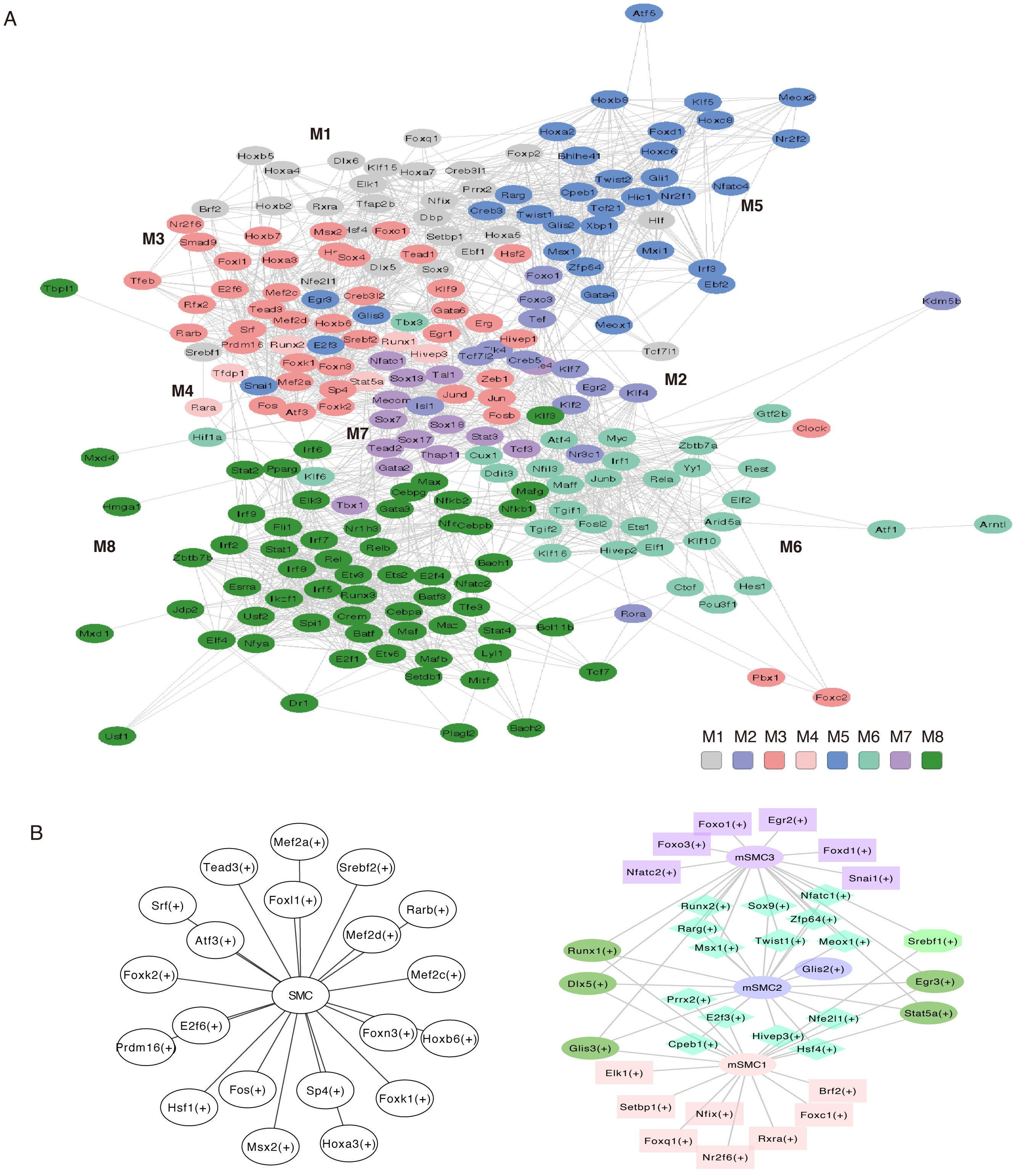

Supplementary Figure 7. A. Regulon association network based on CSI matrix (CSI > 0.8) with different colors representing different regulon modules. B.Network displays the top20 cell type specific regulons for SMCs and mSMC subtypes. SMCs, smooth muscle cells; mSMCs, phenotypically modulated SMCs; CSI, connection specificity index.
